# As we may think and be: brain-computer interfaces to expand the substrate of mind

**DOI:** 10.3389/fnsys.2015.00053

**Published:** 2015-04-14

**Authors:** Mijail D. Serruya

**Affiliations:** Department of Neurology, Thomas Jefferson UniversityPhiladelphia, PA, USA

**Keywords:** brain-computer interface, neurotechnology, neuroprosthetic

Over a half-century ago, the scientist Vannevar Bush explored the conundrum of how to tap the exponentially rising sea of human knowledge for the betterment of humanity. In his description of a hypothetical electronic library he dubbed the memex, he anticipated internet search and online encyclopedias (Bush, 1945). By blurring the boundary between brain and computer, BCI could lead to more efficient use of electronic resources (Schalk, 2008). The advantage of the well-designed direct interface is not simply the discarding of a cumbersome mouse or keyboard in exchange for whispered thought, but the creation of a new, fundamental language bridging essential brain states to discrete items and functions in computers.

Should we achieve such BCI integration, we would come up against the attentional, multi-tasking and global processing limitations of the brain. Both in terms of overall spatial architecture and in moment-to-moment engagement of the world, we appear to have a limited amount of real-estate or bandwidth to work with (Müller et al., 2003; Busse et al., 2005). Just as a stroke may take away a person's ability to do something- such a perceive half the world, or be able to speak—so too one might wonder whether adding on to the brain, at a direct biological level, might provide us with new abilities. We could expand the substrate of the mind itself rather than merely interfacing it to external computers. Components of brain-computer interfaces (BCI) could be re-arranged to create brain-brain interfaces, or tightly interconnected links between a person's brain and ectopic neural modules (Serruya and Kahana, 2008). Such modules—whether sitting in a bubbling Petri dish, rendered in reciprocally linked integrated circuits, or implanted in our belly—would mark the first step on to a path of breaking out of the limitations imposed by our phylogenetic past (O'Doherty et al., 2011; Deadwyler et al., 2013; Vidu et al., 2014). Constructed properly, this system could allow us to experience sensations and movements here fore only granted to other animals—perceiving in true infrared or ultraviolet rather than false-color extrapolations—and we could begin building an architecture to interface with abstract data forms, and indeed with other people, otherwise not possible in 2015. We could extend our nervous systems beyond being a puppeteer of individual vehicles toward being a conductor of swarms of robots, flocks of mechanical birds and fish to change shape and form at our will. Just as vision, sight and touch have their own dedicated neural pathways, we could create novel “search organs” to navigate the internet or large databases, to “feel” molecular structures or social network information.

While we can learn to pay attention to multiple things simultaneously, there appears to be an upper limit to our moment-to-moment information processing capacity after which performance on any given sub-task breaks down (Busse et al., 2005; Dugué et al., 2014). Our brains operate as if having a single attentional spotlight for conscious perception—even if multiple items may be continuously processed in parallel in the unconscious background, reaching conscious perception only when called upon or relevant (Müller et al., 2003; McAlonan et al., 2006). The use of shortcuts or macros are ubiquitous in computer use; by recording a complex series of steps and providing a rule, a macro can allow a computer to blindly repeat the steps and free the human operator. Yet the problem is precisely that the computer is blind: if a file name or operation does not precisely fit a predefined grammar, the performance will grind to a halt, and the previously liberated computer user will have her spotlight forced back to illuminate the problem at hand. A primary goal of expanding the neural substrate will be to enable the brain-computer hybrid to conduct these computerized macro tasks with great speed and efficiency and with just enough conscious awareness of context and content to enable them to proceed. One way to achieve this might be to create a hierarchy of attentional spotlights, or miniature conscious selves, all subsumed by the primary conscious self that one identifies as oneself. One may imagine having avatars: quasi-independent replicas of your own mind created to perform tasks that require a minimum of conscious attention, and which, once trained, could operate without your conscious awareness. These sub-selves, or avatars, could be implemented in tiny constructs of bioengineered autologous neural tissue directly linked to our brain. Just as an individual mouse has his own tiny consciousness devoted to his innumerable mouse tasks, so too could a mouse-brain-sized module of neural tissue be designed to perform the kind of “mindless” computer chores that we would rather not relegate to our primary conscious self. Arrayed with a chain of these interlinked mini-selves, we could entrain each to perform complex tasks that required this minimum of conscious attention, and assign priority flags to which module would know to alert the next level of consciousness up that more attentional consciousness resources were needed for the assigned task. Such abilities to navigate and access information might speed translational science efforts and push the boundaries of human knowledge in an unprecedented manner.

If we can identify the neural signatures of meditative states then we can, both with traditional techniques of breathing and posture ubiquitous to prayer and meditation the world over, and with neurofeedback facilitated by neural modules designed to allow the conscious mind to gain unprecedented perception of the power spectral and ensemble unit firing activity patterns of the brain itself, move our brains into meditative states with improved attentional and integrative capacity (Dickenson et al., 2013; Astrand et al., 2014; Steiner et al., 2014; Strenziok et al., 2014; deBettencourt et al., 2015). By learning to perceive and control the aspects of our brain that give rise to conscious effort and distress we could likewise steer our internal state toward compassion and equanimity; a seamless computer-brain hybrid could be trained to identify when too much information was overwhelming, to steer us to tasks or priority flags that would be best integrated, and also to lay off such automation entirely and let the naked brain be itself. Truly integrated at multiple levels into the brain, these sub-selves- and super-selves born of interfaces linking multiple people—would sleep and dream along with us, and perhaps enter other states of consciousness to promote exchange of information, integration of a unitary consciousness, and the promotion of self-awareness, self-discipline, critical thinking, and compassion.

## Conflict of interest statement

The author declares that the research was conducted in the absence of any commercial or financial relationships that could be construed as a potential conflict of interest.
